# Saffron Aqueous Extract Inhibits the Chemically-induced Gastric Cancer Progression in the Wistar Albino Rat

**Published:** 2013-01

**Authors:** S. Zahra Bathaie, Hamidreza Miri, Mohammad-Ali Mohagheghi, Manijeh Mokhtari- Dizaji, Amir-Ali Shahbazfar, Hadi Hasanzadeh

**Affiliations:** 1 Department of Clinical Biochemistry, Faculty of Medical Sciences, Tarbiat Modares University, Tehran, Iran; 2 Department of Biology, Faculty of Sciences, Zabol University, Zabol, Iran; 3 The Cancer Institute, Imam Khomeini University Hospital, Tehran University of Medical Sciences, Tehran, Iran; 4 Department of Medical Physics, Faculty of Medical Sciences, Tarbiat Modares University, Tehran, Iran; 5 Department of Pathology, Faculty of Veterinary Medicine, University of Tabriz, Tabriz, Iran

**Keywords:** Anticancer, Crocus sativus, Flow Cytometry, LDH, MNNG, Saffron

## Abstract

***Objective(s):*** Gastric cancer is the first and second leading cause of cancer related death in Iranian men and women, respectively. Gastric cancer management is based on the surgery, radiotherapy and chemotherapy. In the present study, for the first time, the beneficial effect of saffron (*Crocus sativus* L.) aqueous extract (SAE) on the 1-*Methyl-*3*-nitro-*1*-nitrosoguanidine* (MNNG)-induced gastric cancer in rat was investigated.

***Materials and Methods:*** MNNG was used to induce gastric cancer and then, different concentrations of SAE were administered to rats. After sacrificing, the stomach tissue was investigated by both pathologist and flow cytometry, and several biochemical parameters was determined in the plasma (or serum) and stomach of rats.

***Results: ***Pathologic data indicated the induction of cancer at different stages from hyperplasia to adenoma in rats; and the inhibition of cancer progression in the gastric tissue by SAE administration; so that, 20% of cancerous rats treated with higher doses of SAE was completely normal at the end of experiment and there was no rat with adenoma in the SAE treated groups. In addition, the results of the flow cytometry/ *propidium iodide staining* showed that the apoptosis/proliferation ratio was increased due to the SAE treatment of cancerous rats. Moreover, the significantly increased serum LDH and decreased plasma antioxidant activity due to cancer induction fell backwards after treatment of rats with SAE. But changes in the other parameters (Ca^2+^, tyrosine kinase activity and carcino-embryonic antigen) were not significant.

***Conclusion:*** SAE inhibits the progression of gastric cancer in rats, in a dose dependent manner.

## Introduction

Gastric cancer is the second major causes of leading death in the most area of the world especially in the East Asian countries (1-4). According to the recent investigations, it is in the first order of incidence between men (26.1 per 10^5^) and second or third (11.1 per 10^5^) order in women in Iran (5). Although a decreasing incidence and death rate has been noted in some countries such as Sweden, Netherlands, Denmark, Ireland, Belgium and New Zealand, its incidence is still high in some others, such as China, South America, Eastern Europe, Costa Rica and Japan (3, 4, 6-12). Despite an overall mentioned decrease in gastric cancer incidence, an increase has been observed in the oldest and the youngest groups of human (12). Thus, gastric cancer has remained as a main public health concern.

Stomach carcinomas are morphologically heterogeneous, and have been classified according to histological profile, degree of differentiation, pattern of growth, etc. The morphological heterogeneity of gastric cancer is also affected by the frequency of occurrence of two or more distinct components in individual cases. According to Correa^׳^s model, gastric cancer initiates from precancerous lesions toward adenocarcinoma. The sequence of events are including: chronic superficial gastritis, atrophic gastritis, intestinal metaplasia, dysplasia, adenoma and adenocarcinoma (gastric cancer) (13). The Lauren's classification is one of the most useful ones, distinguishing two main types of gastric carcinomas, well differentiated (intestinal type) and diffuse types, which show different clinico-pathological profiles and often occur in distinct epidemiological settings (6, 14). Intestinal type is more common and is related to environmental and dietary factors but diffuse type has genetic background and is more aggressive. Like other types of cancer, diagnosis of gastric cancer in its early stages is difficult (4). Symptoms, which are usually non-specific, do not reveal until late stages of cancer. In more than two thirds of cases, gastric cancer is diagnosed at an incurable advanced stage (3, 6, 15). Therefore, due to their different precursor conditions and pathologic features, applied strategies for diagnosis and treatment are different.

Common diagnostic approaches are upper gastrointestinal test series, fibrotic endoscopy, biopsy and computed tomographic scans (CT-Scan) (16). Each of them has advantages and limitations in human studies (3, 6, 15). (17-20), but they are not suitable for rat model of cancer. Recently, we used an ultrasound technique for diagnosis and follow up of cancer induction in rat model of gastric cancer (21).

Applied strategies for treatment of this cancer type in patients are surgery, radiotherapy, chemotherapy and radio-chemotherapy. As mentioned above, in spite of the recent developments in treatment of gastric cancer, 5 years survival is less than 20% with the poor prognosis tool (16). Therefore, main goal of the studies on gastric cancer is to inhibit the development of cancer by screening, elimination of its risk factors, treatment of precancerous lesions and eradication of cancer in early stages; as well as prevention of its initiation.

Current studies have shown that dietary factors such as carotenoids and antioxidants play a key role in the development of cancers. Anticancer activity and protective effects of natural products has extensively been studied. Such activity in the saffron and its ingredients against cancer has been also reported (22, 24). Furthermore, recent pharmacological investigations have declared that saffron has radical scavenger property, shows antioxidant activity and reveals anti-tumor effect (22) (24-29). Various medicinal applications of saffron *Crocus sati*v*us *L. both in the ancient time and the modern world is newly reviewed by us during the history up to the present (30). The mechanism of action of saffron is not known, yet; but it was demonstrated that the saffron extract inhibit cellular nucleic acid synthesis with no effect on protein synthesis in tumor cells (23). Our previous studies also showed that saffron and its active components interacted with DNA (31, 32), histone H1 and H1-DNA complex (33, 34) in the *in vitro* studies.

Since the non-toxic and non-mutagenic effect of saffron extract has been reported (24), in the present study, we evaluated the beneficial effects of saffron aqueous extract (SAE) in both prevention and treatment of chemically induced-gastric cancer in rats. Thus, this study is performed in two separate parts. At first, SAE were used as a chemotherapeutic agent against gastric cancer induced by Methyl nitro nitrosoguanidine (MNNG) in male Wistar rats. Then, the role of SAE as a chemopreventive agent in modifying cancer risk due to administration of MNNG in rats was investigated.

## Materials and Methods

***Animal study***

All rats were purchased from the animal center of the Pasteur Institute, Tehran, Iran and were housed five per cage in a room with controlled temperature and humidity. Handling and treatment of laboratory animals were in compliance with guidelines of the Animal Care Committee of Tarbiat Modares University. The ultrasound images of animals were prepared each mount as explained elsewhere (21).

The process of cancer induction was performed using 1-*Methyl-*3*-nitro-*1*-nitrosoguanidine* or MNNG (Sigma Chem. Co.), as previously discussed (8, 35-37). To confirm the cancer induction, both of the ultrasound images during the study and pathological lams after killing the animals were used.

Before killing the animals, the blood samples were collected for assay of anti oxidant capacity of plasma (FRAP), serum calcium and lactate dehydrogenase. After scarification, the stomachs were separated for histopathologic studies, flow cytometric analysis and preparation of tissue extract for the assay of tyrosine kinase activity and carcino-embryonic antigen (CEA) as a tumor marker.

Animal study was performed according to the following process: 50 male Albino Wistar rats, weighing 100-120 grams, after 10 days of acclimatization, the rats were randomly divided into two main groups. Group A (n=10) was the control group while group B (n=40) was the experimental group. Group A were given water as vehicle and group B were given MNNG 100 µg/ml in drinking water ad libitum for up to 40 weeks. Then, rats in group B were randomly divided into 4 groups including: a cancerous group (B1), which received only MNNG and three treated groups (B2 to B4, respectively) was received 100, 150 and 175 mg/kg body weight per day (mg/Kg) of SAE by intra peritoneal (I.P.) injection for 50 days. All animals were sacrificed after 55 weeks.

***Preparation of SAE***

*Crocus sativus L*. stigmas were macerated in distilled water for three days in cool and dark place. The mixture was subsequently filtered and freezed in liquid nitrogen, and then it was dried in freeze drier. Prepared powder preserved in freezer up to use. To prepare the solutions for injection, after weighing, powder was dissolved in distilled water (33).

***Preparation of MNNG and cancer induction***

MNNG was purchased from Sigma Aldrich Chemical Co. MNNG solution was prepared three times per week with distilled water at a concentration of 100 μg/ml. Administration of MNNG in drinking water is a well established method to study this type of cancer (8, 12, 38, 39).

***Lactate dehydrogenase and Ca*** ^2+^ ***determination***

Serum lactate dehydrogenase was assayed using an LDH reagent kit (Pars Azmoon, Tehran, Iran) by spectrophotometer. This kit measured increased absorbance of NADH at 340 nm during the oxidation of lactate to pyruvate.

Serum Ca^+2 ^was determined by using quantitative Ca^+2^ assay kit (Pars Azmoon, Tehran, Iran) through measuring the absorbance of Ca^+2 ^ at 660 nm.

**Table 1:** The pathologic changes in the rat's stomachs in both normal and MNNG treated groups

Pathologic result Groups	Normal tissues	Chronic inflammation	Hyperplasia	Metaplasia	Dysplasia	Adenoma	Adeno-carcinoma
A	5	0	0	0	0	0	0
B1	0	0	1	1	2	1	0
B2	0	0	1	0	3	0	0
B3	1	0	0	0	3	0	0
B4	1	0	1	0	3	0	0

***Preparation of samples for histopathology***

Gastric antrums from the rats were preserved in 10% neutral formalin. Gastric antrum tissues were processed embedded in paraffin, sectioned at 5 μm, stained with hematoxylin and eosin (H&E), and was histopathologically evaluated.

***Analysis of cell cycle status and apoptosis by flow cytometry***

Tissues of rat stomachs were cut into pieces and their epithelial surface were shaved off, tripsinized (30 minutes) and neutralized by FBS, then filtrated through nylon net of mesh 200 to collect cells. Cells were washed twice with cold phosphate-buffered saline (PBS), centrifuged and fixed in 90% cold methanol in PBS for 1 h at 48ºC. After that, the cells were centrifuged at 1100 g for 5 min, washed twice with cold PBS and incubated with RNase for 10 min. Then the cells were chilled over ice for 10 min, stained with propidium iodide for 15 min and analyzed by flow cytometry (FACScalibur, Becton Dickinson, USA). The apoptosis index (AI)= the apoptotic cells/the total cells. The proliferation index, PI= (cells of S and G2M)/(cells of G0/G1, S and G2M). 

***Antioxidant capacity of plasma***

Antioxidant capacity of plasma in prepared samples were analyzed using FRAP method (41).

***Preparation of tissue extract for the assays of tissue protein, CEA and TK activity***

Fresh tissue homogenized with one to four volumes of a prechilled detergent lysis buffer (RIPA buffer) and Centrifuged at 12,000 g for 10 minutes at 4°C. Clear supernatant carefully removed from pellet. This supernatant is total protein extract and stored at –70°C.

Total protein content of stomach samples were measured through Bradford method (42). CEA was measured by the ELISA kit (a sandwich enzyme immunoassay) from Uscn Life Science Inc, Wuhan, China. Tyrosine kinase activity in the prepared samples was assayed using Universal tyrosine kinase assay kit from TAKARA Bio Inc, Tokyo, Japan, that is based on the activity of recombinant *c-Src*.

***Statistics***

Body weight, total protein, antioxidant capacity of plasma, cell cycle status, apoptosis index, serum calcium, CEA assay, Tyrosine kinase assay, LDH assay were compared among animals and analyzed by ANOVA with Tukey multiple comparisons test and paired samples T-test. Nonparametric data were computed by K independent samples with Kruskal Wallis H- and Mann-Whitney U-tests. A *P*<0.05 was considered to be statistically significant.

## Results

**Figure 1:**
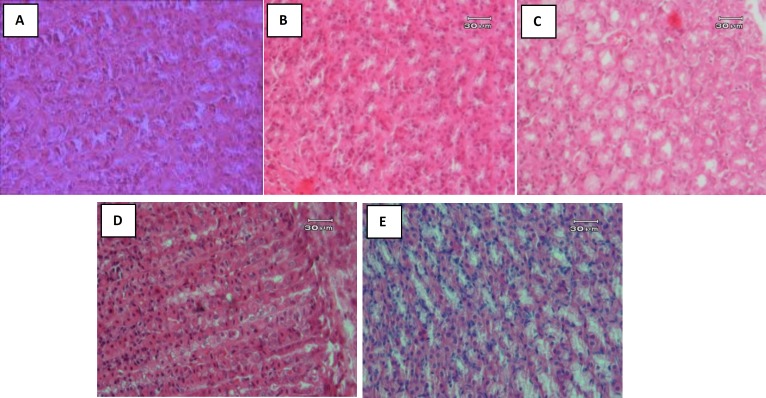
Histopathologic pictures of stomach of all groups of animals in the study. (A) Normal rat stomach. (B) MNNG-administered rat that show hyperplasia: thickening of stomach glandular mucosa and enhancement of cellular population in gastric glands. (C) MNNG-administered rat that show metaplasia: with mucus-producing cells, presence of goblet cells in stomach that is completely abnormal. The mucus producing cells in stomach are hyperplastic too. (D) MNNG-administered rat that show dysplasia: characteristic difference in shape, size, color and dimension of glandular cells plus increase in number and population of them. (E) MNNG-administered rat that show adenoma: with polymorphonuclear cells, increase in cell population, no mitotic figures and no other signs of malignancy like necrosis, hemorrhage, etc. was observed. The cells are well differentiated and nuclei are not that much active, most of them have heterochromatin, but nucleoli are not obvious

Figure 1 (A to E) shows the result of H & E staining of sections of the stomach tissue after sacrificing the rats. As it is seen various pathological changes from hyperplasia, metaplasia, dysplasia and adenoma were observed in the tissue after 40 weeks of MNNG administration. No evidence of metastasis and/or invasion was observed in necropsy. Table 1 summarizes the obtained results of pathological investigation. Here, hyperplasia was not included in gastric cancer classification.

Represented data belong to the rats that their ultrasonic observations during the study were confirmed by their pathologic observations and were survived up to the end of survey. As explained above, number of animals in all groups were equal; however, up to the end of experiment with consideration to the yield of cancer induction and mortality of animals due to MNNG administration, five rats (n= 5) were examined in each of the normal group and control group (only treated with MNNG) and four rats (n= 4) were considered in each of the MNNG-treated groups under treatment with SAE.

There were no significant differences between the body weight of animals in the control and treated groups in the early and end phases of the study (data not shown). However, a significant (*P*<0.05) difference in the body weight of animals in the groups A and B_2,_ before and after treatment was observed.

***Evaluation of effect of SAE on the cell cycle status***

**Figure 2:**
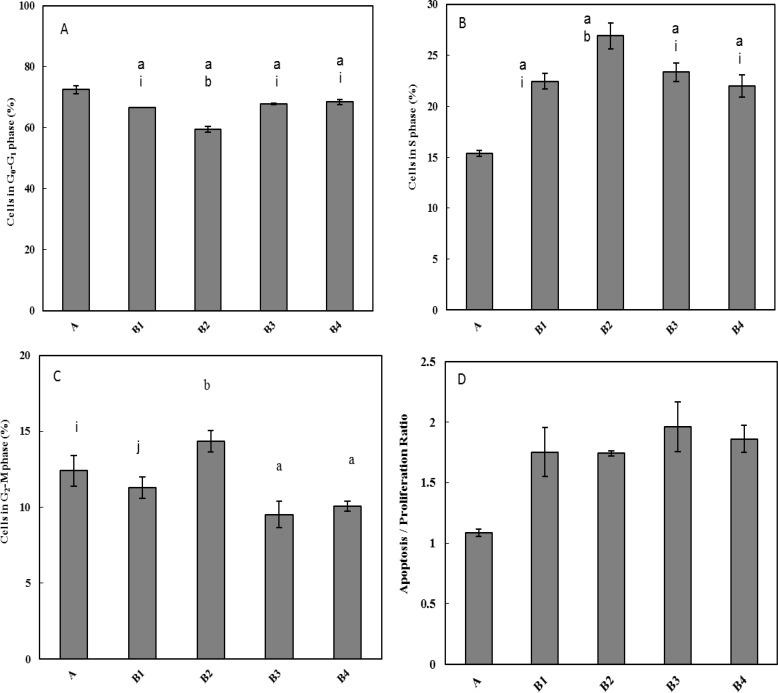
Effect of MNNG administration and SAE treatment on the cell cycle status of the stomach tissue of rats that was determined by flow cytometry. (A). Percentage of the cells placed at G0/G1 phase in different groups. (B). Percentage of the cells at S phase. (C). Percentage of the cells at G2/M phase. a = significant difference between group A with labeled groups; b = significant difference between B_1_ with labeled group; i = significant difference between B_2_ with labeled group, j = significant difference between B_3_ with labeled group. (D). Apoptosis Index/ Proliferation Index (AI/PI) ratio. AI/PI ratio in group A was lower than other groups and there was a significant difference (*P* <0.05) between these groups

Effect of SAE on the cell cycle stages, cell proliferation, apoptosis extent, and apoptosis/proliferation ratio was evaluated by Flow Cytometric analysis and PI staining. Obtained data were shown in Figures 2A-2D. These changes indicated the disruption of the normal cell cycle status and alteration in the AI/PI ratio due to the MNNG administration that was inversely changed by increasing concentrations of SAE, e.g. distribution of the cells in the S phase.

***Antioxidant capacity of plasma***

Effect of SAE on the antioxidant capacity of plasma were measured by FRAP assay. Antioxidant capacity was higher in the normal group (A) than other groups. It means that MNNG administration and cancer induction reduced the antioxidant activity in the plasma. However, it was significantly (*P*<0.05), and in a dose dependent manner, increased after SAE administration. So that, a significant (*P*<0.05) difference was also observed between this parameter in the B2 and B4 groups that was received 100 and 175 mg/Kg SAE, respectively.

***LDH release in blood samples***

Serum LDH levels of the animals in the control and MNNG-treated groups were measured before treatment with SAE and at the end of experiment. Represented results in Figure 4 shows that the LDH level in the serum of MNNG- treated rats (B1-B4) was significantly (*P*<0.05) higher than the normal group (A) before treatment with SAE; and its increase was continuing up to the end of experiment in the MNNG-treated group with no other treatment (B1). However, according to the represented data in this Figure, LDH level was significantly (*P*<0.05) decreased after SAE treatment in the B2-B4 groups. The differences between the LDH levels in these groups and the control rats in the B1 group was also significant (*P*<0.05).

**Figure 3:**
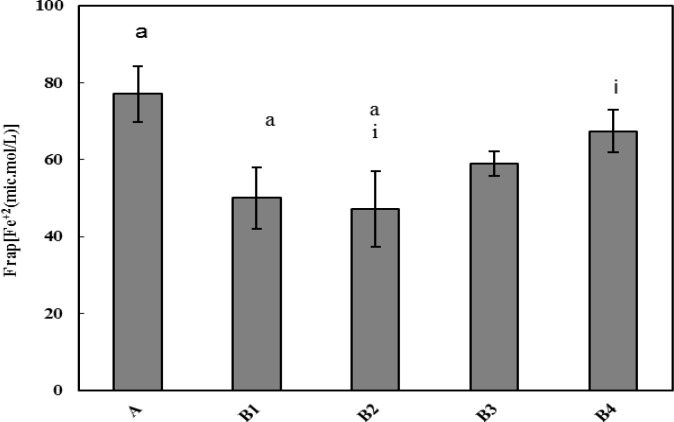
Antioxidant capacity of the plasma of all rats at the end of experiment, which was measured by FRAP method. The significant differences between groups are shown as follows: a = significant difference between group A with B1 and B2; i= significant differences between B_4_ with B2

**Figure 4:**
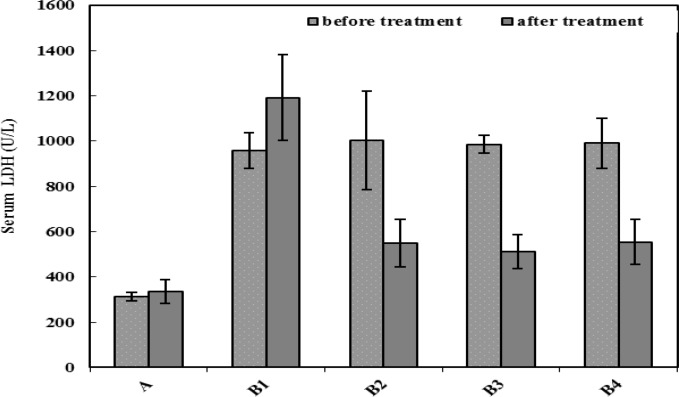
LDH activity in the serum of animals before and after treatment with MNNG and/or SAE. There was a significant difference (*P*<0.05) between group A with other groups. The significant difference (*P*<0.05) was also exist between group B1 with other groups at the end of experiment. The significant difference (*P*<0.05) between serum LDH activity of each group before and after SAE treatment was also observed

**Figure 5:**
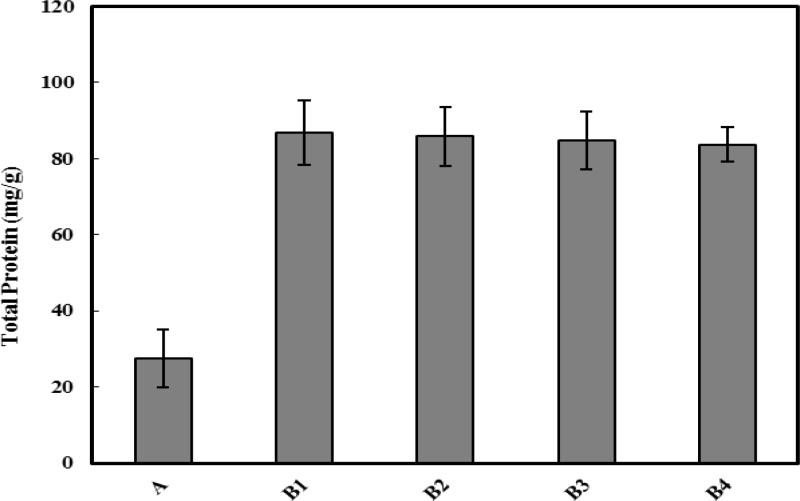
Evaluation of the total protein content of stomach tissues in different groups of rats. Protein content in groups B1 to B4 was significantly (*P*<0.05) higher than group A. All represented data are the Mean±SD of the protein content of tissues on mg/g of stomach

***Total protein determination***

To evaluate the effect of SAE on the protein synthesis, total protein was determined in the extract of 1 g of stomach tissue of animals, by Bradford method and the data showed in Figure 5. As depicted in the Figure, protein content of the tissues was significantly (*P*<0.05) higher after MNNG administration and cancer induction. This increase was continuing even after treatment with different doses of SAE.

***Other parameters***

There were some changes between the normal and cancerous animals in the CEA level, Tyrosine kinase activity and serum calcium (Ca^2+^) before and after treatment with MNNG and SAE, but these changes were not significant (data not shown).

## Discussion

Gastric cancer is a global health problem that has high morbidity and mortality. It is divided into two main types: as intestinal and diffuse types (1, 14, 37, 42). Treatment with surgery followed by chemotherapy and radiotherapy are the method of choice, today; but they could not improve the survival rate and quality of life a lot. Primary prevention, by control of modifiable risk factors and increased surveillance of persons at increased risk, is important in decreasing morbidity and mortality of this harmful disease (3). The inhibitory effects of several chemicals or herbal components in experimental carcinogenesis have been reported (9, 37, 38, 43, 44) (22, 45-48). In continue to our previous studies on the anticancer effect of saffron and its constituents (49), we decided to evaluate the usefulness of saffron aqueous extract (SAE) on treatment of gastric cancer in the model rats.

MNNG was used for induction of gastric cancer in rats. MNNG has been known as a monofunctional alkylating agent (8, 50, 51) with several advantages in comparison with other carcinogens. They are including: a) relatively high specificity for induction of gastric adenocarcinoma when given orally in drinking water; b) with no side effect on rats; c) relatively high percentage of tumor induction. All rats, before and after MMNG administration, was investigated using ultrasound, by the method has been explained in our previous paper (21). Then they were divided into different groups as mentioned above. As the results showed, all lesions induced by MNNG arose at the midpoint of the lesser curvature of the stomach, similar to that reported by others (52-55). The observed alterations in the rats' stomachs were ranged from atrophic inflammation to adenoma (Figure 1).

According to the Lauren's classification two main types of gastric carcinomas, well differentiated (intestinal type) and diffuse types, has been defined. In the diffuse variant, single cells or poorly cohesive clusters of cells infiltrate the gastric wall, often leading to widespread thickening and rigidity of the gastric wall, known as linitis plastica. On the H & E staining, it appears as an empty vacuole and the cells may have the appearance of a signet ring (14). Similar changes in the histopathologic figures in the present study (Figure 1) confirmed the induction of the diffuse type of stomach cancer in rats. The sequence of events observed here is very similar to that reported previously (13, 43). The results of the pathologic examination in Figure 1 and Table 1, indicate that SAE treatment (150 and 175 mg/Kg) significantly decreased the histological severity of the lesions induced by MNNG in the gastric mucosa.

Apoptosis is an important issue in biomedical research of the cancerous cells. The life span of both normal and cancer cells within a living system is substantially affected by the rate of apoptosis. Thus, the chemopreventive and chemotherapeutic agents, who can modulate apoptosis, may be able to affect the steady-state cell populations. Therefore, study of the balance between cell apoptosis and cell proliferation is of great importance for maintaining gastric mucosal integrity. The extent of apoptosis was quantified by flow cytometric analysis of the cells labeled with propidium iodide. The cell cycle perturbations were also examined. Results showed that both AI and PI of the control group were increased, indicating that the balance of the cell apoptosis and proliferation was interrupted by the MNNG administration. However, after SAE treatment these indexes were increased and were higher than in the cancer group without treatment (Figures 2A to 2D). Since apoptosis, cell proliferation, and AI/PI are correlated with histological severity, it seems that SAE with the dose of 150 and 175 mg/kg were effective in treatment of chemical-induced stomach cancer in rat.

It has been well known that the free radical production and subsequent oxidative stress play an important role in the tumor initiation, promotion and progression (37, 56, 57). On one hand, numerous free radical generators have been demonstrated to act as tumor promoters (58). On the other hand, antioxidant agents are believed to protect against cancer by scavenging reactive radical species, resulting in a reduced level of radical-mediated DNA damage (37). A growing body of evidence indicate that carotenoids possess anticarcinogenic, anti-mutagenic and immunomodulating effects (23). According to the data on Figure 3, control group had the highest value of plasma antioxidant capacity (FRAP) among the groups under study. MNNG administration reduced this parameter; but SAE treatment improved the antioxidant capacity of plasma, especially in the groups B3 and B4, receiving higher dose of SAE. 

Cell injury was monitored by measuring the LDH released in the blood. Our results confirmed the MNNG-created cell injury in the stomach. Similar result has been obtained by Mei *et al* (37). As it is seen in Figure 4, at the initial stage of treatment, LDH level in MNNG-received animals (groups B1-B4), was significantly higher than normal one (group A). Also at the last phase of study, due to cancer development, LDH level in the B1 group is higher than other MNNG-received animals (B2-B4) and normal animals (group A) and there was significant difference between them and control group. Our data showed that SAE treatment is effective in decreasing the severity of cancerous alteration in the stomach of rats.

Evaluation of total protein in the stomach tissue was made for

Weight loss is one of the most frequent symptoms reported in the stomach cancer (16, 60), however it is often seen as the late signs of tumor progression (3). According to the obtained data (data not shown), there were only a significant difference between body weight of control and the group treated with 100 mg/Kg of SAE at the initial and final phases of the study. Similar changes in the body weight were also reported by others (8, 43). In addition, this result is compatible with the pathologic data that indicated no adenocarcinoma in rats.

The non-significant differences in the concentration of Ca^+2^ of blood sample before and after treatment is in accordance with the data obtained by Mei *et al* (37). It means that calcium assay is not a proper test for early detection and fallow up of gastric cancer in the treatment studies. Since any of the rats in our study was not received to the last stage of gastric cancer or adenocarcinoma, thus changes in the body weight and serum calcium were not significant.

There were also no significant differences in the CEA level among different groups in this study. It is well known that CEA level is elevated only in one-third of patients, particularly those with large metastatic tumors and in two-thirds of those with well-differentiated intestinal-type tumors. In addition, tumor markers have no preoperative role in the stomach cancer. Such markers may help to identify inoperable, well-differentiated cancers only for preoperative stratification (61). Therefore, the unchanged value of CEA in the present study is completely predictable and consistent with the other results.

It has been shown that MNNG may influence the tyrosine kinase activity as well as the phosphorylation of EGFR through its interaction with EGFR (39). Since protein tyrosine kinases are the enzymes activated as a consequence of the signaling pathway activate by EGFR, and alteration of protein tyrosine kinase are often associated with the uncontrolled cell growth and tumor progression (62), in the present study the activity of TK were determined in the samples. The data indicated that although the TK activity in the normal rats and in the rats receiving SAE was lower than cancerous group without treatment, but these changes was not significant. These findings are consistent with that reported by others (63-66).

In conclusion, our results indicated the gastric cancer induction in the male Wistar Albino rats using MNNG administration. Pathologic study indicated the beneficial effect of SEA on treatment of cancerous rats in the dose dependent manner. Cell cycle study using flow cytometry showed the apoptosis induction in the gastric cancer tissue due to administration of higher doses of SAE. Among different biochemical tests and parameters, antioxidant capacity of plasma, serum LDH level and total protein in the tumor tissue were significantly changed due to SAE treatment.
